# Prevalence and risk factors of *Strongyloides stercoralis* in haemodialysis in Cochabamba, Bolivia: a cross-sectional study

**DOI:** 10.1186/s12882-023-03074-9

**Published:** 2023-02-07

**Authors:** Nicolas Tebib, Nathalie Tebib, Mauricio Paredes, Rosario Castro, Stéphanie Baggio, Mario Villarroel Torrico, Amilcar Alejandro Flores Leon, Maya Herbas Zamorano, Francois Chappuis, Laurent Getaz

**Affiliations:** 1 Intensive Care Unit, Centre Hospitalier de Bienne, Bienne, Switzerland; 2 Division of Internal Medicine, Centre Hospitalier de Bienne, Bienne, Switzerland; 3 Centro de Hemodiálisis de La Media Luna Roja de Irán, Cochabamba, Bolivia; 4 Division of Infectious Diseases, Hospital Clınico VIEDMA, Cochabamba, Bolivia; 5 Division of Prison Health, Geneva University Hospitals and University of Geneva, Geneva, Switzerland; 6 Institute of Primary Health Care (BIHAM), University of Bern, Bern, Switzerland; 7 Labimed, University Mayor of San Simón, Cochabamba, Bolivia; 8 Division of Nephrology, Hospital Clinico VIEDMA, Cochabamba, Bolivia; 9 Division of Tropical and Humanitarian Medicine, Geneva University Hospitals and University of Geneva, Geneva, Switzerland

**Keywords:** Strongyloides stercoralis, Renal dialysis, Chronic kidney failure, Hyperinfection syndrome, Disseminated strongyloidiasis

## Abstract

**Background:**

Strongyloidiasis is an infectious disease that can be fatal in immunocompromised patients. Patients with end-stage renal failure who are on dialysis have a considerably weakened immune system, and organ transplantation is a major risk factor for severe strongyloidiasis. Knowledge of the local epidemiology in tropical and subtropical areas is an essential prerequisite for designing an appropriate strategy to prevent this potentially lethal complication. In this study, we aimed to estimate the prevalence and associated risk factors of *S. stercoralis* infection in patients on dialysis in Cochabamba, Bolivia.

**Methods:**

A cross-sectional study was carried out among patients undergoing haemodialysis in Cochabamba (elevation 2,500 m, temperate climate), collecting information on socio-demographic, lifestyle, and clinical variables, and using one coproparasitological technique (the modified Baermann technique) and one serological (ELISA) test for *S.stercoralis* diagnosis.

**Results:**

In total, 149 patients participated in the study (mean age = 51.4 years, 48.3% male). End-stage renal disease was predominantly (59%) of hypertensive and/or diabetic origin. The positive serological prevalence was 18.8% (95% CI: 13.3%–25.9%). Based on the sensitivity and specificity of the ELISA test, the estimate of the actual prevalence was 15.1% (95% CI: 9.4%–20.7%). Stool samples of 105 patients (70.5%) showed a coproparasitological prevalence of 1.9% (95% CI: 0.52%–6.68%). No potential risk factors were significantly associated with *S. stercoralis* infection.

**Conclusions:**

We found a high seroprevalence of *S. stercoralis* in Bolivian patients undergoing haemodialysis in Cochabamba. We recommend presumptive antiparasitic treatment at regular intervals to avoid the potentially fatal complications of severe strongyloidiasis.

## Background

Strongyloidiasis is an infectious disease caused by the parasite *Strongyloides stercoralis*, a soil-transmitted intestinal nematode. One of the most neglected tropical diseases [1, 2], strongyloidiasis has an estimated global prevalence of around 8.1%, corresponding to 614 million infected people [3]. The parasite is endemic in tropical and subtropical areas, though its presence has also been reported in more temperate climates [4, 5].

The particularity of *S. stercoralis* is its replication capacity within the human body. The larvae, having reached maturity in the intestine, are able to penetrate the colonic mucosa or the skin at the level of the perianal area, resulting in autoinfection, and a persistent infection that can last for several decades if left untreated [4, 6, 7]. In the immunocompetent host, the infection is predominantly asymptomatic. However, in cases of immunosuppression, the process of autoinfection may escape the host’s control, leading to massive parasite replication and hyperinfection syndrome (involving the lungs and gastrointestinal tract) or disseminated strongyloidiasis (with dissemination to other organs such as the brain, kidney and/or liver). Both conditions, which can be summarised as severe strongyloidiasis (SS), carry a mortality rate up to 60–85% [7, 8]. The prescription of corticosteroids is the most common cause of immunosuppression that can lead to hyperinfection syndrome or disseminated strongyloidiasis, even sometimes reported with short-term treatments (< 5 days) and with doses as low as 20 mg of prednisone [8–10]. Other risk factors include organ transplantation, chemotherapy for cancer, haematologic malignancies, infection with human T cell lymphotropic virus type 1, HIV-1, diabetes mellitus, alcohol abuse, and severe malnutrition [7, 8].

Many patients with chronic renal failure on dialysis present a considerably weakened immune system with decreased proliferation of T lymphocytes, especially of the Th2 subpopulation essential for the elimination of *S. stercoralis* [11, 12]. More generally, infections represent the second leading cause of death (after cardiovascular disease) in haemodialysis patients [13]. Compared to the general population, mortality in haemodialysis patients is tenfold higher for pneumonia and approximately 250-fold higher for sepsis [14, 15]. Kidney transplantation, the only treatment currently available to these patients, is a major risk factor for hyperinfection syndrome and disseminated strongyloidiasis [8].

To plan the implementation of preventive strategies for this potentially lethal complication (e.g., early treatment of strongyloidiasis carriers), knowledge of the local epidemiology is an essential prerequisite. This context is especially important in the treatment of frail patients, like haemodialysis patients, or before organ transplantation. In addition, treatment of uncomplicated infection is highly effective and well-tolerated [16]. Unfortunately, epidemiological data are largely lacking in low- and middle-income countries such as Bolivia, where such infections are likely to occur and to remain underdiagnosed and undertreated [4]. Thus, the aim of this study is to estimate the prevalence and associated risk factors of *S. stercoralis* infection in dialysis patients in Cochabamba, Bolivia.

## Method

### Study design

We conducted a cross-sectional study between January and August 2019 in two dialysis centres in the urban region of Cochabamba: the “VIEDMA” public university hospital and the “Media Luna Roja de Iran” dialysis centre. Cochabamba is located 2,500 m above sea level in an inter-Andean valley and has a temperate and semi-arid climate.

### Study population and inclusion/exclusion criteria

The inclusion criteria were to be 1) over 18 years of age and 2) on dialysis for more than three months in one of the two dialysis centres during the study period. The only exclusion criterion was inability to provide consent.

### Laboratory investigation procedures

Clean plastic containers and wooden applicator sticks were distributed and participants were asked to bring two stool samples to the parasitology reference laboratory (VIEDMA) at an interval of at least three days. A specialised laboratory assistant processed and examined the stool samples. This technician had been trained in standardised stool analysis techniques at the Cayetano Heredia Institute of Tropical Medicine in Lima, Peru. The technician was blinded to serological results. Both stool samples were analysed with the modified Baermann technique, chosen in light of its superior sensitivity to other methods, as previously described [4].

For serologies, the sera were separated from the freshly collected blood and stored at -20 ° before the test. IgG was detected by ELISA, and the procedures recommended by the manufacturer (Bordier Affinity Products) were strictly followed. This commercially available test detects Strongyloides IgG antibodies by using somatic antigens from larvae of *Strongyloides ratti.*

### Data collection

We collected information on socio-demographic, lifestyle, and clinical variables by using structured questionnaires determining respondents’ gender, age, level of education, and living area (rural/urban). Other data collected concerned the likely aetiology of end-stage renal disease, history of abdominal pain or diarrhoea within the previous month, history of immunosuppressive medication within the previous three months, frequency of outdoor barefoot walking, and alcohol use.

### Statistical analyses

#### Associations with risk factors

Bivariate analyses (simple logistic regressions) examined the association between potential factors and positive serology. *P*-values < 0.05 were considered significant. We used Stata 16 to conduct the statistical analysis.

#### Estimation of prevalence

Taking into account the performance of the serological test used, we estimated the actual prevalence within the study population. Using a robust methodology, Bisoffi et al. assessed the sensitivity (91%) and the specificity (94%) of the serologic test Bordier-ELISA for the detection of *S*. *stercoralis* in a composite population of subjects from tropical areas and co-infected with other parasitic infections [17]. We calculated the actual prevalence using the following formula: AP = (OP + Sp—1) / (Se + Sp—1), where AP is the actual prevalence, OP is the observed prevalence from positive serological test results found in our study, and Sp (94%) and Se (91%) are the estimates of specificity and sensitivity [17]. The lower and upper 95% confidence interval of the actual prevalence was also computed.

### Ethical considerations

All patients were adults (> 18 years old), personally informed of the purpose of the study and provided informed written consent for participation. All methods were performed in accordance with relevant guidelines and regulations. The study protocol was consistent with the ethical principles of the Helsinki Declaration and was approved by the ethics committee of the University of San Simon (Cochabamba). Participants who tested positive for a parasitic infection (coproparasitological and / or serological tests) were treated free of charge with standard care including a dose of ivermectin (200 μg / kg) for *S. stercoralis.*

## Results

**Fig. 1:**
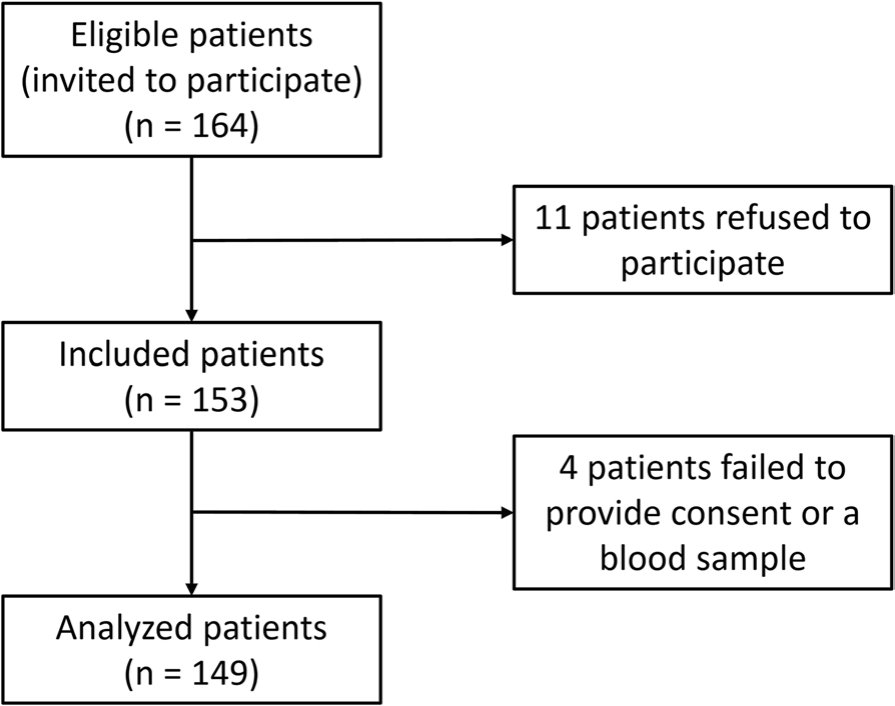
Study flowchart

Of the 164 patients invited to participate in the study, 149 were included in the final analysis. Eleven patients refused to participate and four were excluded (Fig. 1). Of these, two withdrew their consent during the study, one was incapable of discernment and therefore incapable of informed consent, and the fourth was transferred to another dialysis centre before any test could be performed.

The average age of the participants was 51.4 years and 48.3% were men. Table 1 presents the socio-demographic and lifestyle characteristics of all patients. Clinical characteristics and comorbidities are reported in Table 2. Participants’ end-stage renal disease was predominantly of hypertensive or diabetic origin. Only nine (6%) patients had received immunosuppressive therapy during the previous three months. Regarding clinical features, 54 (36.2%) and 53 patients (35.6%) reported diarrhoea or abdominal pain, respectively, in the three months preceding recruitment.

**Table 1:** Socio-demographic and life style characteristics of patients undergoing haemodialysis, Cochabamba, Bolivia

	**Number of patients (%)**
Age (mean, SD)	51.4 (15.7)
Gender	
* Male*	72 (48.3)
* Female*	77 (51.7)
Education level	
No schooling	4 (2.7)
* Primary*	63 (42.3)
* Secondary*	59 (39.6)
* Technical*	6 (4)
* University*	17 (11.4)
Living area	
* Rural*	34 (22.8)
* Urban*	84 (56.4)
* Both rural and urban*	31 (20.8)
Walks without shoes	
* Never*	91 (61.1)
* Sometimes*	49 (32.9)
* Frequently*	9 (6)

**Table 2:** Clinical characteristics and immunosuppressive treatment, patients undergoing haemodialysis, Cochabamba, Bolivia

	**Number of patients (%)**
Probable etiology of renal failure	
* Diabetes*	42 (28.2)
* Hypertension*	40 (26.8)
* Diabetes and hypertension*	6 (4)
* Lupus*	9 (6)
* Other*^*a*^	19 (12.8)
* Unknown*	33 (22.2)
Immunosuppressive drug (last 3 months)	
* Yes*^*b*^	9 (6)
* No*	140 (96)
Abdominal pain (during last month)	
* Yes*	53 (35.6)
* No*	96 (64.4)
Diarrhea (during last month)	
* Yes*	54 (36.2)
* No*	95 (63.8)

^*a*^* Other etiology*: urinary tract infections (5), preeclampsy (4), agenesia (3), obstructive (2), drug toxicity (2), post-streptococcal glomerulonephritis (1), kidney cancer (1), polycystic kidney disease (1)

^b^ Immunosuppresive drugs: corticoids (7), corticoids + azathioprine (2)

**Table 3:** Socio-demographic, lifestyle, and clinical factors associated with *S. stercoralis* in bivariate analyses in patients undergoing haemodialysis, Cochabamba, Bolivia

	n	**Pos**	**Neg**	**OR (95% CI)**	**p**
Age					
< *50 years old*	84	8 (12.3%)	64 (87.7%)	0.45 (0.17–1.09)	.07
≥ *50 years old*	65	20 (23.8%)	57 (76.2%)		
Gender					
* Male*	72	13 (18.1%)	59 (81.9%)	.91 (0.39–2.10)	.82
* Female*	77	15 (19.5%)	62 (80.5%)		
Education level					
* Primary*	67	13 (19.4%)	54 (80.6%)	1.08 (0.46–2.48)	.86
* Secondary-technical-university*	82	15 (18.3%)	67 (81.7%)		
Living area					
* Rural*	65	14 (21.5%)	51 (78.5%)	1.37 (0.59–3.17)	.45
* Urban*	84	14 (16.7%)	70 (83.3%)		
Walks without shoes					
* Never*	91	17 (18.7%)	74 (81.3%)	.98 (.42–2.34)	.97
* Sometimes/frequently*	58	11 (19.0%)	47 (81.0%)		
Abdominal pain (during last month)					
* Yes*	53	10 (18.9%)	43 (81.1%)	1.01 (.41–2.73)	.99
* No*	96	18 (18.7%)	78 (81.3%)		
Diarrhea (during last month)					
Yes	54	9 (16.7%)	45 (83.3%)	.8 (.32–1.91)	.61
* No*	95	19 (20.0%)	76 (80.0%)		

The positive serological prevalence was 18.8% (95% CI: 13.3%–25.9%). Based on the sensitivity and specificity of the ELISA test, the estimate of the actual prevalence was 15.1% (95% CI: 9.4%–20.7%). Of the patients included in the final analysis, 105 (70.5%) provided two stool samples. Two patients presented at least one positive test on the modified Baermann technique stool analysis, corresponding to a coproparasitological prevalence of 1.9% (95% CI: 0.52%–6.68%). Of note, both patients with a positive coproparasitological result had also a positive serological test.

Table 3 summarises the factors potentially associated with a positive serology for *S. stercoralis*, according to simple logistic regressions. There were no significant associations with barefoot walking, rural life, and a low level of education. In addition, the presence of diarrhoea or abdominal pain during the previous three months was not associated with a higher probability of having a positive serology. There was a marginally significant trend towards an increase in serological prevalence for patients 50 years of age or older.

## Discussion

This study demonstrates that strongyloidiasis is widely present among patients undergoing haemodialysis in the city of Cochabamba, with a seroprevalence of 18.8% (estimated actual prevalence of 15.1%). These results confirm the high seroprevalence (22%; estimated actual prevalence of 18.8%) found by Getaz et al. in other patients at high risk of complications living in the Cochabamba region [4].

As previously mentioned, SS is a complication with high mortality risk [7, 8]. Its incidence is likely underestimated due to the lack of recognition of this syndrome. According to Buonfrate et al., given the considerable number of case reports of SS published in non-endemic countries, it is likely that fatal cases of SS are also common in endemic countries but, unfortunately, not published [8]. In addition, between 7 and 12% of cases are diagnosed only at the time of autopsy, which is rarely performed in low and middle income countries [8, 9].

Although haemodialysis or end-stage renal disease as specific risk factors for severe strongyloidiasis have never been investigated, these patients have a weakened immune system and infections represent their second leading cause of death [13]. In particular, sepsis is a very common complication in dialysis patients, with a cumulative incidence of up to 11.7% and a risk 26 to 50 times higher than in the general population [15, 18, 19]. Similarly, septic shock is a complication present in up to 57.3% of patients with SS due to the massive penetration of larvae into the intestinal mucosa that can promote bacterial translocation [9, 20].

In addition, nine (6%) patients received corticosteroid treatment during the three months before recruitment, a major risk factor for SS [7, 21, 22]. Diabetes was identified in 48 (32%) patients, another risk factor although of lesser importance [23, 24].

In view of the high prevalence found in our study and the fatal consequences of delayed diagnosis, it is essential to systematically screen for and treat SS in the event of sepsis or respiratory and/or gastrointestinal symptoms. Furthermore, prevention of SS is of paramount importance in the management of *S. stercoralis*. In groups of patients at high risk for SS and with a prevalence greater than 10%, experts advise presumptive treatment with ivermectin to prevent the life-threatening consequences of strongyloidiasis and in light of this drug’s proven safety [4, 25].

Considering both the chronic immunodeficiency associated with end-stage renal disease and the actual prevalence of 15.1% found in our study, treatment at regular intervals seems to be an appropriate strategy for haemodialysis patients living in an endemic area, such as Bolivia; however, to date, no prospective study has been carried out to validate this approach.

Transplantation, particularly kidney transplantation, is a major risk factor for severe strongyloidiasis [7, 26]. For the aforementioned reasons, any transplant patient living in or coming from Bolivia should benefit from prophylactic treatment with ivermectin before a transplant and then again at regular intervals to prevent a potentially fatal complication.

Our study has several strengths. To our knowledge, this is the first study to determine the prevalence of *S. stercoralis* in haemodialysis patients. Another strength is the high participation rate in the serological study, with only 11 refusals amongst the 164 patients invited to participate. In contrast, our study has also several limitations. First, due to the limited sample size, risk factors of *S. stercoralis* infection may have been missed, such as those identified in previous studies: living in a rural area, walking barefoot, having a low level of education, or having gastrointestinal symptoms [4, 27–29]. Second, collecting stool samples was very difficult in our study, due to low acceptance of this test amongst patients and despite full cost coverage. In addition, these techniques require obtaining fresh stool, which must be sent in to the laboratory for analysis no more than four hours after collection for living larvae to be detectable therein. Most of the patients in our cohort lived more than two hours from their dialysis centre, making the delivery of fresh stool samples difficult or even impossible. The high discrepancy between the actual prevalence and the coproparasitological prevalence (15.1% vs. 1.9%) could be linked to a delay in the delivery of numerous samples and to the known limited sensitivity of coproparasitological techniques. Nevertheless, despite a low prevalence in the parasitological method, seroprevalence is high. It is likely that a large majority of patients with a positive serology are, in fact, infected with S.stercoralis but undiagnosed with the Baermann method. Since these patients can evolve to a SS, treatment is recommended. Finally, serological testing is a diagnostic tool with suboptimal performance, which is why an actual prevalence calculation is presented. The actual prevalence takes into account the possibility of false-positive results due to cross-reaction with other helminths, as the sensitivity and specificity of the ELISA were assessed in a composite population including subjects co-infected with other parasitic infections [16]. Also, The risk of false-positive results due to immunological memory is low since more than two-thirds of those treated have a negative serological test for strongyloidiasis within a few months [30]. Furthermore, the sensitivity reported in the literature was measured in populations that were predominantly not immunosuppressed. It is possible that the sensitivity of serological testing is even lower in dialysis patients, whose ability to synthesize antibodies could be reduced, similar to their altered response to hepatitis B or influenza virus vaccines [31]. Thus, this potential bias could lead to an underestimation of the real prevalence of strongyloidiasis in the study population.

These limitations related to diagnostic tests are important to consider and reinforce the proposition for presumptive prescription of antiparasitic treatment to all patients at high risk of complications.

## Conclusion

This study reveals a high prevalence (15.1%) of S. stercoralis in Bolivian patients undergoing haemodialysis in Cochabamba, a city located at approximately 2,500 m above sea level and benefitting from a temperate semi-arid climate.

Therefore, presumptive antiparasitic treatment should be prescribed at regular intervals to avoid the potentially fatal complications of severe strongyloidiasis. This is especially important in the context of the inherent immunodeficiency of haemodialysis patients, the suboptimal sensitivity of serological screening, the difficulty in collecting stool samples of good quality, and the high safety of ivermectin treatment. These recommendations should also apply before and after kidney transplantation for patients residing in or coming from Bolivia.

Moreover, it is essential to systematically identify and treat severe strongyloidiasis without delay in the event of sepsis or respiratory and/or gastrointestinal symptoms in haemodialysis patients from endemic areas.

## Data Availability

The datasets used and/or analyzed during the current study are available from the corresponding author upon reasonable request.

## Abbreviations

SS: Severe strongyloidiasis
HIV-1: Human immunodeficiency virus 1
